# Career choice of tourism students in a triple-whammy crisis

**DOI:** 10.1371/journal.pone.0279411

**Published:** 2022-12-27

**Authors:** Monica Waichun Choy, Alexander Seeshing Yeung

**Affiliations:** 1 Faculty of Management and Hospitality, Technological and Higher Education Institute of Hong Kong, Hong Kong Special Administrative Region, Hong Kong, People’s Republic of China; 2 Institute for Positive Psychology and Education, Australian Catholic University, North Sydney, New South Wales, Australia; The Hong Kong Polytechnic University, HONG KONG

## Abstract

Hong Kong hospitality and tourism industry has been battered by the triple whammy of social unrest, Sino-US trade war and COVID-19 pandemic in recent years. To understand how vulnerable tourism students may be in terms of career shock when facing the three major challenges, 407 tourism students in Hong Kong were surveyed. Structural equation modelling found a positive correlation between affect (an intrinsic, motivating factor) and extraneous events (an extrinsic, demotivating factor), indicating that motivation and demotivating factors may co-exist. Affect was more positively correlated with three career choice outcomes (intent to join the industry, desire for a lifelong career, and resilience in face of unfavourable circumstances) than was Extraneous. In face of career shock arising from extraneous events, tourism students still tend to have a strong intent to join the workforce, take it as a lifelong career, and remain resilient despite the hardship. However, the career shock was a greater concern for those in hotel-related disciplines and for students aged over 20 than younger ones. The findings offer an empirical basis to guide policy makers, academia and the industry in strategy formulation to ensure sustainable quality and manpower supply in the post-crisis future.

## Introduction

The years of the 2020’s are, not only an ‘era of challenge and change’, as Floyd [1] puts it, but also an era of crisis. Among higher vocational education sectors, hospitality and tourism (labelled as tourism hereafter) is one of the most vulnerable under unexpected circumstances such as security crises, natural disasters, economic/financial crises and health safety crises [2]. This study examines intrinsic and extraneous factors affecting tourism students’ career choice in response to career shock [3] arising from a recent triple-whammy crisis [4] in Hong Kong.

In Hong Kong, hit by the triple whammy of social unrest, Sino-US tensions and COVID-19, tourism-related industries recorded a 15-year high unemployment rate of 10.6% well beyond the onslaught of SARS [5]. Higher education institutions providing vocational programs in tourism have experienced immediate declines in enrolment in relevant courses [6]. Despite continual manpower shortage prior to the pandemic outbreak [7], an upended employment climate and uncertain industry outlook have been disrupting school leavers’ intention of joining the industry after graduation [8], sowing the seeds of manpower crisis that is envisaged to impede tourism industry revival. We may anticipate an undersupply of talent and competition for manpower among tourism organizations post-COVID-19 [9,10]. Compounded with the recent emigration wave out of Hong Kong, the industry might have to face a double squeeze of human capital reduction at both operative and managerial levels, which can have detrimental effects on the industry’s sustainable quality and growth [11]. This is a major concern for Hong Kong where tourism is a key pillar of the city’s economy. Hence, it is prime time for tourism education providers and industry practitioners to proactively prepare for the talent shortages in the aftermath of this crisis era [12].

One’s career choices could be influenced by motivating and demotivating factors, sometimes referred to as ‘push’ and ‘pull’ factors. Within Lashley’s [13] push-pull conceptualization, Davidson and Wang [14 p238] defined push factors for hospitality staff turnover as those “that cause an employee to leave”, and pull factors as “attractions associated with working elsewhere”. Likewise, Ali Shah, Fakhr, Ahmad, and Zaman [15] also suggested that employees’ turnover intentions are influenced by internal controllable push factors (e.g., attitude and job satisfaction) that trigger one’s withdrawal intention and external uncontrollable pull factors (e.g., renumeration package offered by other organizations to attract new employees). Meanwhile, Zgheib [16], examining career choices also using a push-pull conceptualization, defines push as uncontrollable forces (e.g., organizational restructuring and declining career prospects) and pull as voluntary (e.g., desire for growth and status), which contradicts with the generally known assumptions of pull as uncontrollable factors and push as voluntary factors. Interestingly, while these researchers define push and pull somewhat differently, common across them is an emphasis on the co-existence of unique internal and external, motivating or demotivating, factors that influence one’s decision making. Also common across them is the notion of an internal, self-oriented factor versus an external, less controllable factor, which may drive one’s decision to achieve a goal or deter one’s action to do so. Hence, while a positive internal attribute (e.g., intrinsic interest) drives an individual to persist and set self-directions in a career, negative, extraneous factors (e.g., an undesirable work environment) may trigger avoidance tendencies and an intention to quit [17].

It is therefore the complex interplay of these perceptions that create work valence and influence vocational education students’ career choice [18]. For educators, considering that individual intrinsic factors are malleable while extraneous contextual factors are not, individual factors are particularly important factors to consider as they can be changed through appropriate intervention. By 2022, the first cohort of Generation Z (Gen Z) born between 1995 and 2009 will become the new wave of workforce [19]. Given Gen Z holds divergent expectations, needs and self-perceptions as compared with the preceding generational cohorts [20], a mismatch between supply and demand, or needs and expectations, may become an obstacle that deters new blood from joining the industry. Given the labour-intensive nature of tourism, massive loss of staff during COVID-19 has led to emerging manpower shortage which deters the sustainability of the industry, especially when tourism workers are most needed when the industry has a chance to revitalize from the pandemic. Identification of the factors that drive students to make their initial career choice and lifelong commitment, and the barriers thereof is vital. Studies about career intention and choice of Hong Kong tourism students have been limited. While career intention in tourism is influenced by intrinsic and extraneous factors (or a combination of push and pull factors), unclear is the association between these factors among Hong Kong generation Z students amid an era of triple whammy of social unrest, Sino-US trade war and COVID-19 pandemic, hence the rationale for the current study. Understanding the voice of the Gen Z undertaking tourism programs about the determinates of their early and long-term career choice is vital for educational institutions and tourism industry leaders to ensure a sustainable future workforce during this unprecedented time. As extant studies have called for more empirical research on the combined impacts of multiple crises in different contexts [2,21], to enlighten this uncharted area, the overall quest of the present study is to investigate how intrinsically motivating and extraneously demotivating push/pull factors drive and hinder tourism students’ initial career choice in the wake of the triple-whammy crisis (i.e., social unrest, Sino-US tensions and COVID-19). In specific, the study aims to examine (1) the role of affect (intrinsic personal interest) as a motivating factor and extraneous events (external uncontrollable happenings contributing to the triple-whammy crisis) as a demotivating factor in career choice and other career-related outcomes, and (2) identify any differences in the motivating and demotivating drivers and career outcomes across individual and contextual background variables. This work contributes to existing knowledge of behavioural intention literature by exploring how push and pull factors facilitate tourism students’ career intention, or prevent them from joining the industry after graduation, which underlie their initial career motivations. We also aim to provide a deeper insight into the heterogenous responses from students of different seniority and disciplines of tourism studies about the facilitators and barriers in shaping their occupational aspirations. The findings thus have the potential of identifying a desirable approach to facilitating students’ career choice for tourism and detecting what student and program characteristics may need attention to nurture future talents and match post-COVID-19 manpower needs. Given the vulnerability of the industry, understanding tourism students’ concerns will enlighten us of similar issues in other disciplines more broadly [22]. The evidence-based knowledge generated from this study may also improve graduate employment rates, achieving an optimal balance between supply and demand.

### Essential motivational factors

#### Affect—An intrinsic factor

‘Affect’ here refers to one’s affective self-concept, which is the belief one holds about one’s feelings, attitudes and emotions [23]. Affective self-concept is an influential intrinsic motivational force for one to engage and thrive in a specific task or behavior. It is essential for the sustainability of the industry because a positive Affect leads to positive emotional responses [11] and long-term engagement [23,24]. On one hand, tourism research has shown that a negative attitude towards working conditions and industry prospects are the main reasons for not choosing the industry [9,25]. On the other hand, vocational interest and satisfaction in challenging jobs are known motivators that counter the industry’s structural barriers (e.g., low pay, anti-social working hours, and vulnerability to external environments) that prevent individuals from joining the workforce or staying with the organization [10,26]. Hence, we would expect students who find interest in the tourism program they attend to have a higher tendency to join the industry and to stay there for a long time.

#### Extraneous events as a career shock

Negatively affecting one’s career choice are numerous factors, career shock is one among many demotivating factors. Career shock is defined as unanticipated, extraordinary, and exogenous events beyond one’s control that trigger employees to reconsider their future career [27], or drive their career-related behavior such as career choice upon course completion [28], decisions on further education or temporary exit from the industry [29], and change of employment status [30]. Career shock is manifested in one’s response to the strength of the events that are characterized by their novel, disruptive and critical nature for sensemaking [31]. Induced by multiple contrasting intrinsic/motivating and extrinsic/demotivating factors, the magnitude, frequency, manageability, continuity and valence of career shock may vary across individuals and circumstances in a specific career field context [27]. While the current pandemic situation is one of these factors, other barriers include social unrest, violence, war, international disputes, political instability, economic crisis and natural disaster, etc. are counted [2]. New university graduates could experience precarious employment and financial insecurity for up to 5 years in their early career, graduating into an unfavorable socio-economic condition as a result of career shock [32].

In Hong Kong, the social unrest that started from 2019 [33], followed by COVID-19 in 2020, has left Hong Kong ‘living in uncertainty’ [34]. For Hong Kong, any international affair may have an impact on the city. The South China Sea dispute [35], and the ongoing trade war between the USA and China, which has impacted global economy [36], are inevitably affecting Hong Kong as part of China. While COVID-19 may be regarded as a major demotivating extraneous factor contributing to career shock that deters individuals from joining the tourism industry [3,10,29,37], the co-existence of multiple crises simultaneously is rare. The cumulative impacts resulting from the scarcely happened triple-whammy crisis appeared to have severely disrupted Hong Kong’s tourism industry since 2019, and have changed employee behavior and decisions as to join, stay with, or exit from the industry [10]. This career intention dilemma could be regarded as an entry shock or career shock event for the tourism students and industry practitioners respectively [37,38]. Although entry shock or career shock may elicit negative outcomes (e.g., lowered career choice intention), extant studies show that the effect of shock on tourism students’ career choice outcomes may vary from country to country. For example, tourism students in India [39] and Ecuador [40] are optimistic about tourism rebound, opting to pursue a career in tourism despite the uncertain industry outlook as the COVID-19 pandemic grinds on. Similarly, New Zealand tourism students adopt situational career strategies in the short and medium term but remain assured and committed to join the industry in the long run [29]. On the contrary, the passion to join tourism industry has been diminishing among tourism students in Mainland China [41] and Philippines [42] amid the pandemic outbreak. For Omani tourism students, Atef and Al Balushi [43] found that tourism is considered as a stepping stone to launch their career; hence a small percentage of them plan to make it a lifelong career. Previous researchers have discussed how COVID-19 influences occupational attitude of tourism students [37,41,42], but no work has been dedicated to examining the incremental impacts of multiple crises on tourism students’ career choice in Hong Kong context. It is of interest to know whether the pessimism found in Mainland China during COVID-19 holds true in Hong Kong, a city within China but features an East-meets-West culture.

### Essential career outcomes

#### Intent to join the career and lifelong career choice outcomes

Career aspirations may be differentiated into immediate and long-term aspects. The immediate aspect is the intent to join the career after training [44,45], while lifelong career choice stresses a continuing career decision across the lifespan from graduation to retirement [43]. The industry needs new recruits to join the workforce while continual employment of existing staff is crucial for sustainable organization development and the staff working within it. Hence the inclination of graduates to join the industry and stay in the job as long as they can are equally important. Research has shown that motivating factors such as how competent one feels about oneself and how much one likes the career both influence their views about future opportunities and career [11]. There are also demographic factors that may play a significant role in career choice [44]. Existing studies have shown that students’ first career choice is influenced positively by intrinsic/motivating factors such as career interest [8,11,46], expectations and self-efficacy beliefs [47] but negatively influenced by demotivating factors such as undesirable remuneration, job nature, and career prospect [9,48]. A recent study on Hong Kong travel trade industry practitioners revealed that factors such as high customer contact, anti-social working hours, poor working environment, supervisor and co-worker relations, remuneration, and career prospects are the key demotivating factors that trigger job change [10]. Nevertheless, such destabilizing factors of job change are interrelated and not necessarily caused by career shock that are characterized as unpredictable, unwelcomed, and uncontrollable [3,27,28]. One of the questions that remains unanswered is how the context of perceived pushes and pulls encourage/discourage students’ initial career choice in tourism, hence, the rationale for the current study.

#### Resilience in the workforce

Resilience is also referred to as ‘buoyancy’ when individuals face hardships and mishaps [49]. Resilience may be regarded as a trait of an individual or a process [50]. One is said to be resilient if one has the ability to bounce back and flourish amid distressing situations [51]. Student resilience reflects their capabilities to cope with adverse environment and challenging events that could be enhanced by internal (e.g., self-efficacy) and external determinants (e.g., school, educational opportunities and work experience) [52–54]. Extant evidence supports a positive link between resilience and career behavioral intention in challenging times, as reported by Bullough [52] in an American study. Extant literature also suggests that cultural and contextual differences may affect individual resilience. For example, a recent study maintained that one’s sense of competence is more strongly related to perceived resilience than is affect towards tourism among students in Hong Kong [11]. A Turkish study showed that gender and program level did not impose significant influence on resilience for high school students [55]. On the contrary, a Finnish study revealed that the higher the education level, the lower the students’ resilience [56]. For vocational education providers, policy makers, and employers, it is essential to have a better understanding of the link between resilience and its internal and external drivers to maintain sustainability of the industry.

### Individual and contextual factors

Apart from the intrinsic and extrinsic factors, other individual and contextual factors may contribute to one’s career choice. Individual factors such as gender and age, and the context in which the student is situated, such as the level and discipline of study are likely to influence career choice. Information about the effects of these contextual factors will provide educators with insights into effective ways to facilitate career choice and outcomes for students of various characteristics and in various contexts.

#### Gender

Studies have shown divided findings on gender differences in career choice. In general, males seem relatively higher in self-efficacy [57], and are more inclined to take risks, but culture also makes a difference. While Kenyan male students are more passionate about tourism [58], American female students have higher career aspirations than males [26]. Existing studies have revealed mixed views, reflecting on different cultural and gender reactions to uncertainties when making decisions [59,60]. In Hong Kong, tourism programs and jobs attract more females than males. Female students generally outnumber male students in term of first job decision in tourism [61] and admission to tourism-related programs [62]. However, females’ representation has been found to slump to 20 percent at the executive level [63]. With this apparent gender bias favoring males, it would not be surprising that males tend to be more optimistic about their future [64].

#### Age

Students’ career preference evolves with age. Older students tend to set clearer career goals [65]. Older students may show less concern about immediate financial success, but strive to achieve higher-level needs (e.g., self-actualization and recognition, etc.) [66]. However, Generations Y and Z may hold different values [20]. Generation Y may be concerned more about manager-subordinate relationship, career prospect, and remuneration package whereas Generation Z values a culturally diverse workplace over salary. Regardless of age, however, long anti-social working hours and potential workplace health and safety risks discourage people from joining the industry [9,20].

#### Program level

In a study in Mainland China, undergraduate students’ intention to pursue a tourism career was found to be remain at less than 20%, regardless of their educational attainment [67]. Another study found that undergraduates and postgraduates were the most reluctant to join the industry due to various negative perceptions (e.g., heavy workload, low pay, and lack of career prospect) [68]. A recent study revealed that academic attainment did not influence students’ career intention to join the industry [69]. To our knowledge, no study has examined career intention among Hong Kong tourism students at different program levels. It is of interest to know whether the findings from Mainland China hold true in Hong Kong, which is part of China but with a more westernized education system. It is also of value to higher education providers and vocational program developers to know which program level would be more sustainable.

#### Program discipline

Among various sectors in the tourism industry, the hotel sector has enjoyed flourishing times prior to the pandemic outbreak and social unrest. While COVID-19 has impacted most tourism-related sectors, hotel business has been one of the most seriously affected since travel restrictions were introduced [70]. Hence, we may envisage that students in a program that is related to hotel business would have greater sensitivity to extraneous factors of the triple-whammy crisis in their career choice.

## The present investigation

The main goal of the current study is to examine how the triple-whammy crisis, which introduces a career shock among Hong Kong tourism students, may influence their career intention and choice decisions. The study has two objectives:

1. To examine the associations of intrinsic (affect) and extraneous (events) factors with a range of career outcomes (i.e., intent to join the career, lifelong career choice, and resilience in the workforce).
2. To identify any differences in the motivational (affect) and demotivational (extraneous) drivers and career outcomes (intent; lifelong; resilience) across individual (gender; age) and contextual factors (program level; program discipline).

**Fig 1:**
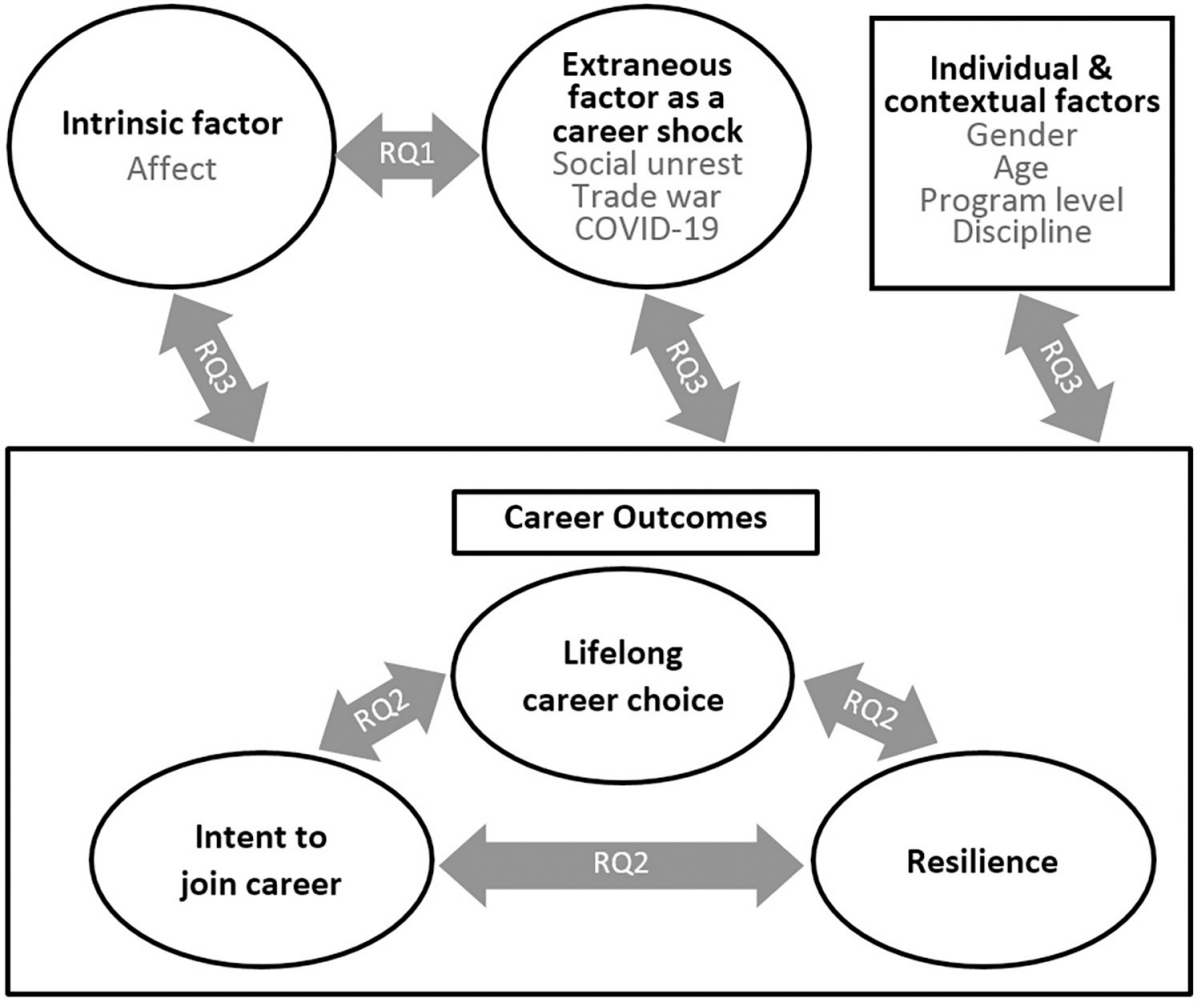
Conceptual framework.

Specifically, the study attempts to answer four research questions (RQs; see Fig 1):

RQ1. How do the intrinsic (affect) and extraneous (events) factors correlate with each other?
RQ2. Can the three career outcomes (intent to join the career, lifelong career choice, and resilience in the workforce) be clearly differentiated from each other?
RQ3. How does affect correlate with the three career outcomes, and what are the correlations of extraneous events with the three outcome variables?
RQ4. Do the two individual characteristics (gender and age) and two contextual characteristics (program level and program discipline) display different strengths in predicting the three career shock outcomes?

## Conceptual framework

The conceptual framework for this research was inspired by the push-pull conceptualization regarding decision making. However, due to the debatable, ambiguous definitions of push and pull factors [13–17], and given our focus on the intrinsic motivational factor of personal affect (personal interest in the industry) and an extrinsic factor of extraneous events, we use the terms ‘intrinsic’ and ‘extraneous’ to highlight the orientations and functions of these factors in career decision making. Fig 1 is a visual representation of the conceptual framework on which basis subsequent statistical analyses are based. On the upper left-hand side of Fig 1 is affect, an intrinsic motivating factor for career choice. The upper-middle of the diagram representing extraneous events that occurred in Hong Kong as a career shock. The upper right-hand side variables are individual and contextual factors. Below the intrinsic and extraneous factors in Fig 1 are a range of career outcomes, which may or may not be affected by the factors above. The outcomes considered here include: intent to join the career, lifelong career choice, and resilience in the tourism workforce.

## Materials and methods

There were approximately 7,700 students enrolled in tourism-related programs in Hong Kong [71] when the research was designed. Students who were studying in tourism-related local full-time undergraduate and sub-degree programs in VPET institutions were invited to complete an online survey (paper copies were provided instead if requested). Purposive sampling was used to select participants who possessed relevant knowledge and experience to achieve the objectives of the research [72]. The survey focused on five constructs: Affect (an intrinsic factor), Extraneous (an external, probably demotivating factor), and short- and long-term outcomes (i.e., intent to join the career, lifelong career choice, and resilience in the workforce) using multiple items on a six-point scale: 1 (Disagree strongly) to 6 (Agree strongly). The survey also collected respondents’ demographic data (gender, age, program level, and program discipline). Most of the measurements were adapted from the literature and were adjusted to suit the study purposes while the measurement for extraneous events was developed by the authors to align with the context-specific environment in Hong Kong. Items that measure extraneous events were related to infectious diseases, international disputes, social unrest and violence, and any global issues that can damage the economy.

As a result, a survey consists of a total of 17 items for 5 constructs was prepared (See Table 1 for constructs, sample items and sources). All the chosen measures have good psychometric properties reported in previous studies and demonstrated good Cronbach’s alphas supporting their reliability and internal consistency (Table 4). The survey was commented by two university professors for content validation. Following university ethics procedures, informed consents were obtained from all participants before they responded to the survey. A pilot study was conducted to fine-tune the instrument before the actual study. A bilingual (English-Chinese) survey was administrated between January and September in 2021, when the triple-whammy impacts were prevalent.

**Table 1:** Survey constructs, sample items and sources.

Construct	No. of Items	Sample item	Source
Affect	3	I am interested in my preferred hospitality discipline.	Yeung, Craven, and Kaur [24]
Extraneous	4	I fear that any infectious disease can suddenly destroy the economy. I fear that people may suddenly lose their jobs when international disputes occur (e.g., political fights, trade war). I fear that social unrest and violence may happen any time causing economic crisis (e.g., anti-government protests). I fear that any global issue can trigger an economic downturn at any time.	Developed by authors
Intent	3	I will join the hospitality workforce after graduation	Yeung and McInerney [45]; Yeung, Kadir, Kuppan, and Foong [44]
Lifelong	3	I hope to have a life-long career in tourism/hospitality	Yeung and McInerney [45]; Yeung, Kadir, Kuppan, and Foong [44]
Resilience	4	I can cope when things don’t go well for me	Martin and Marsh [49]; Pennington, Dillon, Noble, and Yeung [51]

*p* < .001.

Preliminary analysis included descriptive statistics and reliability analysis. Using the statistical package of Mplus [73], we first tested a 5-factor model to examine the factor structure of the hypothesized Affect and Extraneous factors together with career choice outcomes (Intent, Lifelong, and Resilience) (Model 1). Then the four characteristics (gender, age, program level, program discipline) were added (Model 2). Mplus was chosen among other statistical packages for its flexibility in adjusting the syntax to run alternative models and its advantage, as highlighted by Narayanan [74], of simultaneously handling continuous, categorical, observed, and latent variables in a variety of models including confirmatory factor analysis and structural equation models.

Model fit was assessed by the comparative fit index (CFI), Tucker-Lewis index (TLI), and the root mean square error of approximation (RMSEA). The chi-square test statistics are also reported. In general, the values of TLI and CFI equal to or larger than .90 are considered an acceptable fit [75]. The value of RMSEA ranging between .05 and .08 is generally accepted as a close fit to a fair fit [76]. For Model 1, factor loadings showing the relations of each latent construct with each of the observed variables were inspected. We then checked the latent factor correlations showing the associations of the latent constructs, which should be clearly smaller than 1 so as to be differentiated from each other. For Model 2, to identify group differences in terms of gender (coded 0 = male; 1 = female), age (0 = age<20; 1 = age from 20 up), program level (0 = sub-degree; 1 = degree or above), and program discipline (0 = non-hotel-related; 1 = hotel-related), the correlations of the individual (gender, age) and contextual variables (program level, and program discipline) with the five constructs established in Model 1 were examined.

## Results

### Demographic profile

A total of 407 completed surveys were collected (115 males and 292 females; age from under 20 to over 50). The students were studying at various levels of the program (266 in certificate and diploma sub-degree programs; 141 in degree programs) of which 279 and 128 of respondents have been undertaking hotel-related programs (e.g., hotel operations) and non-hotel-related program (e.g., food and beverages; theme park), respectively (See Table 2).

**Table 2:** Descriptive statistics.

		Gender		Age		Program		Hotel-related	
		Male	Female	<20	20 up	Sub-degree	Degree	No	Yes
	*N*	115	292	198	209	266	141	128	279
Affect	Mean	4.32	4.21	4.40	4.10	4.39	3.96	4.36	4.19
	*SD*	(0.89)	(0.96)	(0.87)	(1.04)	(0.90)	(0.96)	(0.83)	(0.99)
Extraneous	Mean	4.22	4.34	4.15	4.46	4.26	4.40	4.04	4.43
	*SD*	(1.13)	(1.03)	(1.04)	(1.01)	(1.00)	(1.16)	(1.06)	(1.03)
Intent	Mean	3.95	3.84	3.94	3.80	3.96	3.70	3.74	3.39
	*SD*	(1.00)	(1.02)	(1.00)	(1.04)	(0.97)	(1.11)	(1.05)	(1.00)
Lifelong	Mean	3.36	3.05	3.33	2.95	3.26	2.90	3.30	3.06
	*SD*	(1.07)	(1.09)	(1.11)	(1.04)	(1.03)	(1.16)	(1.08)	(1.09)
Resilience	Mean	4.14	4.02	4.16	3.96	4.16	3.86	4.08	4.04
	*SD*	(0.79)	(0.80)	(0.85)	(0.73)	(0.68)	(0.94)	(0.85)	(0.77)

### CFA model solution

Model 1 (Table 3) resulted in an acceptable fit (CFI = .938, TLI = .922, RMSEA = .072, χ^2^ = 335.515(109 *df*). The solution is presented in Table 4. All the factor loadings were statistically significant (all>.50). The latent factor correlations were all positive with the largest being .72 between Intent and Lifelong, indicating that all the five constructs were clearly differentiable from each other (*r*<1).

**Table 3:** Models.

	χ^2^	*df*	CFI	TLI	RMSEA
Model 1. Five factors	335.515	109	.938	.922	.072
Model 2. Four characteristics + 5 factors	430.018	157	.931	.907	.065

**Table 4:** CFA solution.

	Affect	Extran	Intent	Life	Resil	Gender	Age	Prog	Disc
Alpha	.90	.86	.87	.86	.73				
Mean	4.24	4.31	3.87	3.13	4.06				
*SD*	(0.94)	(1.06)	(1.02)	(1.09)	(0.79)				
**Factor Loadings**									
Item 1	.83**	.67**	.90**	.79**	.69**	1	1	1	1
Item 2	.89**	.76**	.66**	.89**	.56**	--	--	--	--
Item 3	.89**	.83**	.92**	.77**	.68**	--	--	--	--
Item 4	--	.85**	--	--	.63**	--	--	--	--
Affect	--								
Extraneous	.26**	--							
Intent	.63**	.29**	--						
Lifelong	.59**	.32**	.72**	--					
Resilience	.62**	.34**	.49**	.42**	--				
Gender	-.04	.05	-.04	-.15*	-.09	--			
Age	-.18*	.15*	-.08	-.19**	-.15*	.04	--	
Program	-.23**	.08	-.10	-.18**	-.23**	.04	.51**		--
Discipline	-.10	.19**	.08	-.12	-.03	.10*	.39**	.22**	--

*N* = 407.

*p < .05.

**p < .001.

The alpha reliability estimates were all good (ranging from .73 to .90; Table 4). The mean scores (above the mid-point on a 1–6 scale) indicated that the students were positive in their affect to tourism (*M* = 4.24) but were also concerned about extraneous happenings that could be detrimental to their career (*M* = 4.31). The mean scores for the three outcome variables (Intent, Life, and Resilience) were around the midpoint of the scale (3.87, 3.13, and 4.06, respectively). Between Affect and Extraneous, the correlation was significantly positive (*r* = .26), although lowest among all latent correlations.

The correlations of Affect and Extraneous with the career outcome variables (Intent, Lifelong, Resilience) display a clear pattern of more positive correlations for Affect than for Extraneous. An interesting observation is that even for Extraneous (a presumably demotivating variable) is positively correlated with all three career outcome variables). Intent and Lifelong are found to be the strongest positive correlates (*r* = .72), implying that those who intend to join tourism are also likely to choose it as a lifelong career, which seems reasonable.

Model 2, with individual and contextual characteristics added to the five constructs derived from Model 1, resulted in an acceptable fit (CFI = .931, TLI = .907, RMSEA = .065, χ^2^ = 430.018(157 *df*). Table 2 shows the descriptive statistics by gender, age, program level, and program discipline. For Model 2 (Table 3), the critical considerations are the correlations between the five constructs established in Model 1 (Table 3) (Affect, Extraneous, Intent, Lifelong, and Resilience) and the individual (gender, age) and contextual (program, discipline) characteristics.

As shown in Table 4, the correlation of gender with Lifelong (*r* = -.13) was statistically significant (*p* < .05), indicating that male students (*M* = 3.36) were more likely to choose this industry for a lifelong career than females (*M* = 3.05; Table 2).

The correlation of age with Affect, Extraneous, Life, and Resilience were statistically significant (*r* values = -.18, -.15, -.19, -.15), indicating that younger students tended to score higher in these four constructs (Table 4). Specifically, younger age students tend to like tourism more, feel less concerned about extraneous barriers to their career, choose tourism as their lifelong career, and be more resilient when faced with challenges.

Program level was significantly negatively correlated with Affect (*r* = -.23), Life (*r* = -.18), and resilience (*r* = -.23), indicating that sub-degree students were positive in these three constructs. Specifically, sub-degree students were higher in Affect (*M* = 4.39) than undergraduates in the degree programs (*M* = 3.96; Table 2), Lifelong (*M* = 3.26 vs. 2.90 respectively; Table 2), and Resilience (*M* = 4.16 vs. 3.86; Table 2). In short, sub-degree students were more positive in their affect to learning, choice of tourism as a lifelong career, and resilience in face of challenge.

Differences across hotel-related and non-hotel-related disciplines were small except for Extraneous. Hotel-related students tended to be more concerned about extraneous events (*r* = .19 between Discipline and Extraneous, *p* < .00; Table 4). Specifically, hotel-related students (*M* = 4.43) were more concerned about extraneous events than non-hotel students (*M* = 4.04; Table 2) whereas discipline differences were small and not statistically significant for the other constructs.

## Discussion

Drawing upon the push-pull paradigm of motivation, this study aimed to examine how two presumably competing forces drive or hinder tourism students’ initial career choices in light of the triple-whammy crisis. The analysis attempted to answer four RQs, the findings of which are elaborated below.

### RQ1. Correlation between Affect and Extraneous factors

The correlation between Affect (a motivating force) and Extraneous (a presumably demotivating force) was positive, indicating that these two apparently conflicting factors were not directly opposing forces (which would have been zero or negative otherwise). That is, while one likes tourism, one can also feel the fear arising from extraneous events. Hence, motivating and demotivating factors may co-exist as part of the career. Supporting previous studies is the result showing that one’s affect (i.e., the extent of career interest) is a powerful motivator of career choice that can counter unfavorable job natures [10,26]. The tendency of one joining the industry depends on the relative strength of the motivational forces, although demotivational forces may adversely affect one’s decision [9,25]. Our results show that tourism students in Hong Kong are prone to take positive attitude to embrace challenges that lies ahead. To tourism education providers, evidence shows that it is crucial to enhance students’ passion and positive affect for recruitment and retention for a sustainable workforce. However, the strength and impact of the presumably demotivating force of extraneous factors need to be further investigated in future research. Although the newly introduced Extraneous construct has good face validity and displays good reliability (alpha = .86), interpretation of its relations with other constructs will need further testing, preferably using a between-network approach to construct validation (see Arens, Yeung, Craven, and Hasselhorn [77] for details).

### RQ2. Distinctiveness of intent to join the career, lifelong career choice, and resilience

Overall, the three career outcome variables are positively related but clearly distinguishable from each other. This implies that tourism education providers may promote these outcomes in a distinctive manner. The positive correlations suggest that tourism students who intend to join the workforce may also tend to stay with the industry and endure challenging work environments. Our result supports the finding that a positive correlation was found between resilience and career behavioral intention during challenging times [52]. Our findings also echo with Shad [39] and Ortiz [40] who argue that tourism students are still keen on joining tourism industry even the industry outlook continues to remain fragile amidst pandemic, but do not seem to support Birtch [41] and Benaraba [42] suggestion that tourism students’ career intention is tapered off in the crisis era. In addition, while our findings partly support Reichenberger and Raymond [29] who argue that tourism students’ career choice is contingent upon disruptiveness of external environment in the short to medium term but remain confident in the long run, there is no evidence to support the argument that students in tourism consider the industry only as a stepping stone to employment [43]. The inconclusive findings imply that students’ attitude towards career choice and tolerance for unpredictable events may be industry and culture specific [46,59]. Further research is required to examine the driving forces and barriers for students to make career decisions in different contexts. For this specific sample of students, while their intrinsic interest plays a vital role of drawing them to the career, it seems that they are also aware of the hardships and potential risks arising from extraneous events. After all, they know that such risks are always part of their career, which all practitioners in the field have to face anyway”.

### RQ3. Respective correlations of Affect and Extraneous with the three outcomes

In general, Affect (a motivating factor) was found to be more positively correlated with all three outcomes (intent to join the career, lifelong career choice, and resilience in the workforce respectively) than was Extraneous events (presumably a demotivating factor), which indicates a stronger association of intrinsic motivation with the career choice outcomes. Interesting was the positive association of Extraneous with all three outcome variables. The positive correlations imply that in face of serious challenges arising from extraneous events, this sample of students still have strong intent to join the workforce, take it as a lifelong career, and remain resilient despite the hardship. Similar to previous studies [8,10,11,46], our findings further demonstrate that career interest effectively pre-empt Hong Kong tourism students’ career choice even during the deeply troubled time.

The patterns of associations of the ‘push’ and ‘pull’ forces as motivating and demotivating factors with the career outcome variables found in the analysis have interesting theoretical implications. In the push-pull conceptualizations by various researchers, the push and pull effects may end up in the same direction or in opposite directions, depending on the salience of the constructs and the individual’s interpretation of them. For Davidson and Wang [14 p238], for example, while an intrinsic feeling of dissatisfaction is a push that may “cause an employee to leave”, “attractions associated with working elsewhere” may serve as a pull factor further exacerbating the thought of attrition. Hence in this case, the push and pull forces operate in the same direction for a decision. For Zgheib [16], favorable intrinsic factors pull individuals being an entrepreneur voluntarily while unfavorable extraneous factors push them to leave the present job, which may end up with conflicting drivers of action in opposite directions. In the present study, we adopted the terms used by Ali Shah, Fakhr, Ahmad, and Zaman [15] differentiating between push as a self-related, internal factor (Affect in this case) and pull as an external, less controllable extraneous factor, to avoid confusion in the interpretation of these driving forces and their directional impacts on decision making. After all, the current push-pull conceptualization will benefit from clearer definitions and operational illustrations.

### RQ4. Correlations of individual and contextual characteristics with outcomes

Male students were more likely to take tourism as a lifelong career choice than females. The finding is consistent with Kinyua and Burugu [58] acknowledging Kenyan male students being more passionate about the industry, but is contrary to the findings of Chuang and Dellmann-Jenkins [26] with an American sample. For male students, perhaps due to their relatively higher self-efficacy belief [57] and inclination to take risks, they are more willing to consider tourism as a lifelong career even in this era of crisis. Our findings, together with previous research showing males’ optimism about the future [64] and higher career advancement opportunities [63], not only reinforce gender differences and gender inequality [63], but also highlight the significance of enhancing students’ self-efficacy, especially for females. Our results also provide further evidence that females are more vulnerable in a chaotic and crippled industry environment [78]. Age differences were found in four constructs. Younger students had more positive affect, more willing to choose tourism as a lifelong career, and tend to be more resilient, and were less concerned about extraneous events. It seems that the career shock due to extraneous events was a greater concern for older students (aged over 20 in this sample) than younger ones. Under Hong Kong’s education system, students who are under 20 are mostly in junior form of a sub-degree/undergraduate course while those over 20 are mostly in senior form. Our results showing that extraneous events aroused grave concerns of students aged over 20 may be due to their frustration arising from the effects of a shrunken job market that senior form students are likely to suffer immediately. This has created enormous financial and emotional pressure, complicated also by the growing student loan debt which they need to pay back [79].

Findings of the present study suggest that sub-degree students had higher affect towards tourism industry. The results may be due to the consequences of impressive internship experience [80]. Practical working experience gained from internship and vocational interest is a double-edged sword that can both be a motivator and demotivator of students’ career choice [25]. Favorable internship experiences encourage students to join the industry based on a positive evaluation of industry-person congeniality [69,81], and reinforce self-efficacy [47], and vocational interest [8]. Interestingly, sub-degree students also tended to be more resilient in comparison with counterparts from other program levels. Our findings do not support those studies suggesting that gender and program level did not impose significant influence on resilience [55] but are partly congruent with the notion about a negative association between education level and students’ resilience [56]. Individual, contextual and cultural differences might account for the divergent career behavioral intentions we found here. The favorable resilience found in the sub-degree students in the present Hong Kong study may be due to an optimistic view of the time available to them to pursue a degree and wait for the industry to return to pre-pandemic prosperity. A recent New Zealand study has also shown that tourism students’ perceived valency of pursuing further tourism study remains high during the COVID-19 pandemic [37].

Hotel-related students had stronger concerns about extraneous factors. This is not surprising because hotels rely heavily on external demands traditionally despite the fact that hotel revenue is mainly generated from staycation, long stay residential and quarantine stay in the wake of the pandemic. While the domestic market can support some of the non-hotel sectors (e.g., food and beverages; theme park), hotels cannot operate without visitors. The hotel industry is therefore one of the most vulnerable sectors during difficult times [70]. Higher education students taking a hotel-related major are, not surprisingly, more concerned about extraneous factors than anyone else, and more reluctant in joining the hotel workforce. Similar findings were also reported in previous related studies [41,42].

Overall, the data yielded from this study about the effects of individual and contextual factors provide educators with insights into who needs attention and assistance when it comes to career choices. With this useful information, educators may devise effective ways to optimize the career outcomes of students with various characteristics and backgrounds.

### Practical implications

The present study aimed to investigate how push-pull forces (presumably motivating and demotivating factors) may drive or hinder tourism students’ initial career choice following the triple-whammy crisis in Hong Kong. Our findings highlight the importance of enhancing students’ career interest (affect) to improve their career intention and decidedness to join the tourism industry. Based on the aforementioned findings and discussions, while motivating (i.e., affect) and demotivating factors (i.e., extraneous fear) may co-exist, students tend to be motivated to enter and stay in the industry when they value intrinsic motivating factors (e.g., interest, satisfaction). Evidence shows that career interest is a strong motivating factor to drive students to join the industry in the first place and take tourism industry as a lifelong career. At the macro-level, policy makers should roll out more relief measures to maintain tourism organizations’ continuity and retain existing industry practitioners [82]. Given the global tourism landscape that has been evolving during the ongoing COVID-19 and other exogenous events, Hong Kong need to put its efforts to reinforce the city’s attractiveness as a tourism destination by reformulating its marketing strategies and preparing for international tourism rebound [83]. At the meso-level, more tourism-related subjects may need to be introduced as elective modules in the senior secondary curriculum to familiarize students with the industry and stimulate their career aspirations. Using various formal and informal assessment tools (e.g., personality/ability testing and interview), tourism education providers can help students refine their interests by demonstrating the connections between the learning programs and career opportunities [84]. Once a student has developed a strong passion for the occupation, the intrinsic motivation may outweigh the fear for extraneous events that cause hardship [11,85]. COVID-19 has spurred the adoption of information technology into daily operations in tourism-related industries (e.g., app platform, touchless technology, robotics and artificial intelligence) to boost tourist confidence and reduce operational costs [86,87]. Workplace technology application should be enhanced across the curriculum in tourism programs to prepare students for a career and meet organizations’ and customers’ needs [88]. For vocational orientation programs, voices from industry practitioners carry equal, or sometimes stronger, credibility than those from academia [89,90]. Guest speakers from the industry could be invited to share their real-world experience and insight about industry prospects. Students’ interest can be enhanced when the bond between speakers and students is established. To familiarize students with major hotel features and working environments, on-site/virtual reality tours can be organized [91]. On-site/virtual career fairs can also be held to provide students with opportunities to explore employment pathways, interact and network with potential employers, and submit job applications. In essence, to ensure the industry’s sustainable quality and manpower supply, apart from the government support, it is important for tourism educators to provide students with realistic assessments of their abilities, accomplishments, and interests. Curriculum designers may also consider a revamp of the curriculum to meet the emerging needs of the post-COVID future and ensure transferability of learnt skills/knowledge to rebuild students’ confidence and interest to launch their career after graduation.

## Conclusion

Our study revealed the implications of the triple-whammy crisis as career shock among tourism students in Hong Kong. First, the present study demonstrates the significance of promoting intrinsic motivation to counter a fear of extraneous factors as a career shock/demotivator; second, career choice outcomes (intent to join the industry, lifelong career, and resilience) are positively linked to each other but clearly differentiated, so they may be promoted in distinctive ways; third, the significance of enhancing students’ occupational interest as a strong motivating factor to outweigh perceived extraneous threats to a tourism career; and fourth, the need to promote a gender balance to minimize wastage of human resources. This paper documents a number of key contributions to the field of tourism and hospitality study. According to the author’s knowledge, no published work was dedicated to the influence of the triple-whammy crisis on Hong Kong tourism students’ career aspirations. Our study appears to be the first study examining the magnitude of career shock amidst the consecutive challenges of social unrest, Sino-US tensions and COVID-19 among tourism students that lays the groundwork for further research on other industries and elsewhere. It has also responded to the call for empirical studies on career behavioral intention and reinforced the need for research with samples from different sociodemographic and contextual backgrounds. This study may also redound on policy makers, academia, and tourism industry players in strategy formulation to embrace the post-crisis future. However, due to the cross-sectional nature of the study design, this study cannot determine causal inferences. A longitudinal study will be useful to test causal relations of identified variables. Because the study was conducted in Hong Kong, the findings may not be generalized to other cultures or tourism markets. Given tourism in Mainland China is recovering swiftly from COVID-19 with its domestic demand, researchers may consider comparing the career choice of Gen Z in Mainland China with Hong Kong or other countries. We may anticipate that recruitment competition will be intensified post COVID-19 for new blood and pre-COVID experienced workers. It is of interest to examine employees’ intention to (re-) enter the industry among current and past generations (baby-boomers, Gen X and Y).

## Supporting information

S1 Data (XLSX)
